# Structural transformations and stability of benzo[*a*]pyrene under high pressure

**DOI:** 10.1107/S2052252524010455

**Published:** 2025-01-01

**Authors:** Wenju Zhou, Andrey Aslandukov, Anastasiia Minchenkova, Michael Hanfland, Leonid Dubrovinsky, Natalia Dubrovinskaia

**Affiliations:** a Material Physics and Technology at Extreme Conditions, Laboratory of Crystallography University of Bayreuth 95447Bayreuth Germany; b Bayerisches Geoinstitut University of Bayreuth 95440Bayreuth Germany; c European Synchrotron Radiation Facility CS 40220 38043GrenobleCedex 9 France; d Department of Physics, Chemistry and Biology (IFM) Linköping University SE-581 83 Linköping Sweden; Université de Sherbrooke, Canada

**Keywords:** high-pressure crystallography, molecular crystals, polycyclic aromatic hydrocarbons, phase transitions, benzo[*a*]pyrene

## Abstract

Benzo[*a*]pyrene (B*a*P), C_20_H_12_, is a representative of polycyclic aromatic hydro­carbons (PAHs), which are ubiquitous in nature and the universe, where they are subjected to extreme conditions. This paper reports the results of investigations of the high-pressure behavior of B*a*P up to 28 GPa using *in situ* synchrotron single-crystal X-ray diffraction. We identified two previously unknown polymorphs, B*a*P-II (*P*2_1_/*c*) at 4.8 GPa and B*a*P-III (*P*1) at 7.1 GPa. The structural transformation from B*a*P-I (*P*2_1_/*c*) to B*a*P-II (*P*2_1_/*c*) manifests as an abrupt change in the intermolecular angle and the unit-cell parameters *a* and *b*, whereas the transformation from B*a*P-II (*P*2_1_/*c*) to B*a*P-III (*P*1) is characterized by a decrease in symmetry. According to density functional theory calculations, B*a*P-III is the most stable phase above 3.5 GPa. These studies advance our understanding of the structural dynamics and stability of PAHs under high pressure.

## Abstract 2

This study explores the high-pressure behavior of benzo[*a*]pyrene, revealing two previously unknown polymorphs at 4.8 and 7.1 GPa. These findings enhance our understanding of the structural dynamics and stability of polycyclic aromatic hydrocarbons under extreme conditions.

## 1. Introduction

Polycyclic aromatic hydro­carbons (PAHs) are complex organic compounds consisting of two or more condensed benzene rings. Owing to their exceptional properties and widespread applications, these compounds have attracted significant attention from geoscientists, chemists and physicists (Silinsh & Cápek, 1997 ▸; Farchioni, 2001 ▸; Allamandola *et al.*, 1987 ▸). PAHs are ubiquitous in interstellar space, comprising 20% of the carbon in the universe and potentially representing the most abundant free organic molecules in space (Allamandola *et al.*, 1985 ▸; Ehrenfreund & Charnley, 2000 ▸; d’Hendecourt & Ehrenfreund, 1997 ▸). It is thought that research into the evolution of PAHs under pressure may help us to understand the origins of our universe (Mimura *et al.*, 2005 ▸; Mimura & Toyama, 2005 ▸).

Benzo[*a*]pyrene (B*a*P) is one of the two isomeric species of benzopyrene (C_20_H_12_), a representative of PAHs, formed by a benzene ring fused to pyrene. The compound is abundant (Bukowska *et al.*, 2022 ▸) and can be found, for example, in coal tar as a product of incomplete combustion of organic matter at high temperatures. It is a yellow solid under ambient conditions.

Crystals of B*a*P were first described by Iball (1936 ▸), who obtained them from B*a*P solutions in the form of needles and plates and determined their symmetry and unit-cell parameters using X-ray diffraction (XRD). The needle-shaped crystals were monoclinic (*P*2_1_/*c*) whereas the plate-shaped crystals were orthorhombic (*P*2_1_2_1_2_1_). The crystal structure of the monoclinic B*a*P was later solved (Iball & Young, 1956 ▸) and refined (Iball *et al.*, 1976 ▸). Subsequently, one more ortho­rhombic B*a*P polymorph was reported (Contag, 1978 ▸). However, it was found to be unstable and gradually, within 6 months, transformed into the known monoclinic B*a*P. Low-temperature single-crystal XRD (SC-XRD) measurements at 120 K (Carrell *et al.*, 1997 ▸) enabled a more precise structure analysis.

In this work, we have investigated the behavior of B*a*P in the pressure range from ambient to 35 GPa using synchrotron SC-XRD in a diamond anvil cell (DAC). We have observed two previously unknown high-pressure polymorphs, B*a*P-II and B*a*P-III, and report the results of the analysis of their structures under pressure.

## 2. Experimental

### 2.1. Sample preparation

A crystalline powder of B*a*P of 96% purity was purchased from Merck. Single crystals were selected under an optical microscope and preselected for high-pressure XRD studies in DAC No. 1 at ambient pressure (see Table S1 of the supporting information for a summary of all experiments). The two preselected crystals and a piece of ruby were loaded in the membrane-type DAC No. 2 equipped with Boehler–Almax type diamonds (Boehler, 2006 ▸) with culets 250 µm in size and a rhenium gasket with a hole ∼120 µm in diameter and a thickness of ∼30 µm. He was used as a pressure-transmitting medium. The DAC No. 2 was gradually pressurized from 2.2 to 35 GPa.

### 2.2. SC-XRD experiments

SC-XRD studies at room temperature were conducted in DAC No. 1 and DAC No. 2 on the ID15B beamline (λ = 0.4100 Å, ESRF) with a beam size of approximately 2 × 2 µm. In both experiments a micro-grain of tungsten was placed at the center of the pressure chamber along with the sample. The strong X-ray absorption signal of tungsten was used to adjust the rotation center. The pressure was determined by the ruby luminescence method (Mao *et al.*, 1986 ▸). At each pressure step, the data were collected in step scans of 0.5° on rotating the DAC from −34 to +34° about the vertical axis (ω scan). For single-crystal data analysis (peak search, unit-cell finding and data integration), the *CrysAlisPro* software (Rigaku Oxford Diffraction, 2015 ▸) was employed, and the crystal structures were determined using *SHELX* (Sheldrick, 2008 ▸) and refined utilizing *Olex2* (Dolomanov *et al.*, 2009 ▸). Crystal structure visualization was performed using *VESTA* (Momma & Izumi, 2011 ▸). *EoSFIT7* (Angel *et al.*, 2014 ▸) was used to fit the pressure–volume data.

### 2.3. Theoretical calculations

Our density functional theory (DFT) calculations were performed using the *Vienna Ab-initio Simulation Package* (*VASP*) (Kresse & Furthmüller, 1996 ▸) with the projector-augmented-wave method (Blöchl, 1994 ▸) and the generalized gradient approximation functional was used for calculating the exchange-correlation energy, as proposed by Perdew, Burke & Ernzerhof (PBE) (Kresse & Joubert, 1999 ▸). Additionally, we employed the DFT-D3 method for dispersion correction (Grimme *et al.*, 2011 ▸). The Brillouin zone was sampled with a 7 × 1 × 2 Monkhorst–Pack (Monkhorst & Pack, 1976 ▸) special *k*-point grid for B*a*P-I and B*a*P-II, and 7 × 2 × 1 for B*a*P-III. Furthermore, the valence states 2*s*^2^2*p*^2^ for carbon and 1*s*^1^ for hydrogen were used with the energy cutoff of 520 eV for the plane-wave basis set. The geometries were optimized until the remaining atomic forces were less than 5 × 10^−3^ eV Å^−1^ and the energy convergence criterion was set at 10^−5^ eV.

## 3. Results

On compression of B*a*P-I (*P*2_1_/*c*) to 4.8 GPa in an He pressure medium, we observed a phase transition to a previously unknown monoclinic polymorph B*a*P-II (*P*2_1_/*c*). The next phase transition occurred at 7.1 GPa to B*a*P-III with a triclinic structure (*P*1), which was preserved up to about 28 GPa. At the next pressure step of 35 GPa, the XRD pattern disappeared. Below we describe in detail the structures of all B*a*P polymorphs observed in this work.

**Figure 1:**
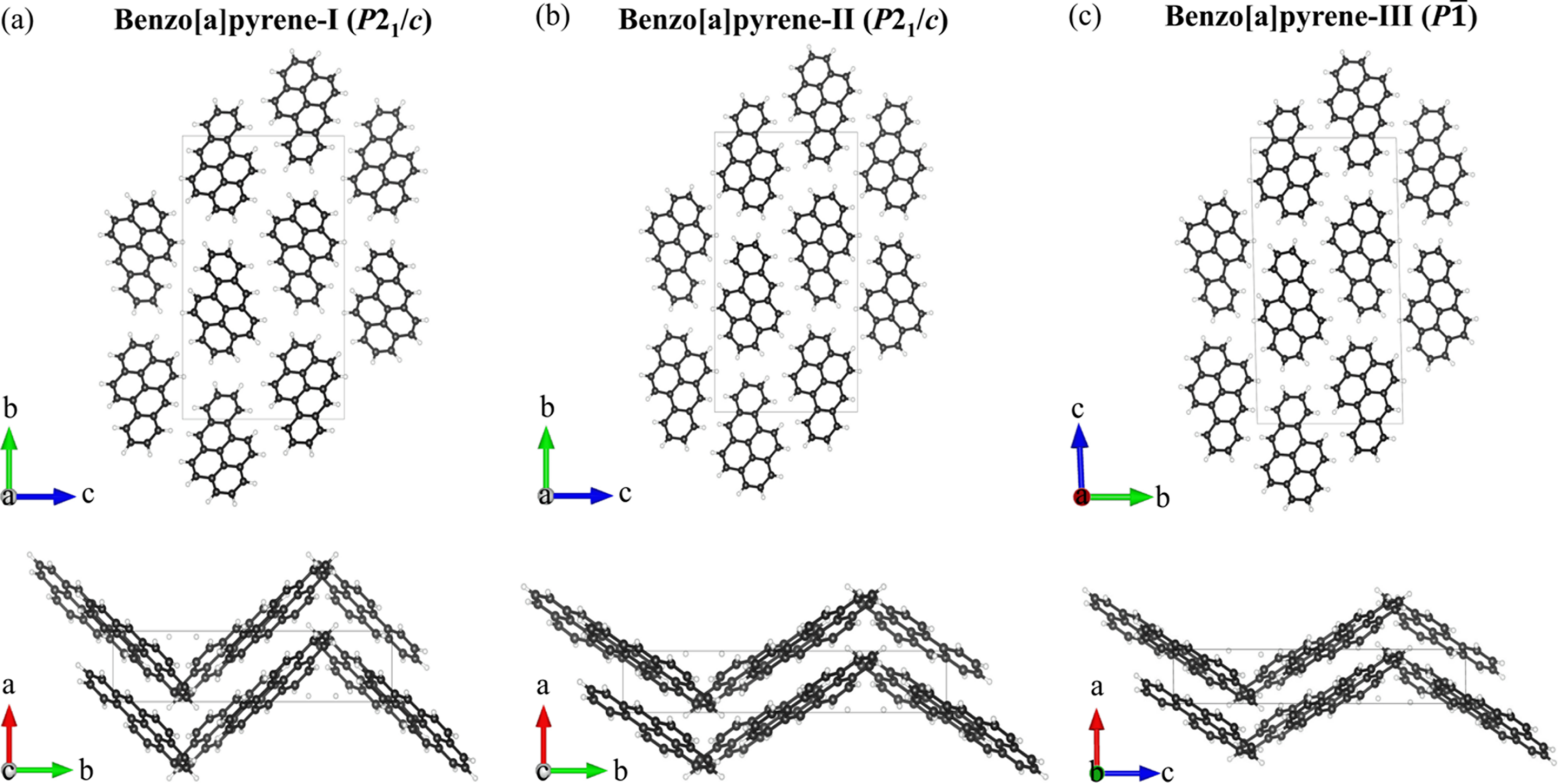
Crystal structures of B*a*P polymorphs. (*a*) B*a*P-I under ambient conditions, as viewed along the *a* (top) and *c* (bottom) axes; (*b*) B*a*P-II at 4.8 GPa, viewed along the *a* (top) and *c* (bottom) axes; (*c*) B*a*P-III at 7.1 GPa, as viewed along the *a* (top) and *b* (bottom) axes: carbon atoms – black, hydrogen atoms – white.

The structure of B*a*P-I determined at ambient and 2.2 GPa [Fig. 1 ▸(*a*)] is similar to the previously reported monoclinic structure (Carrell *et al.*, 1997 ▸). For a detailed comparison with B*a*P-I at 120 K, see Table S2. The structure is monoclinic (space group No. 14, *P*2_1_/*c*) with the following unit-cell parameters under ambient conditions: *a* = 4.5384(3) Å, *b* = 20.439(5) Å, *c* = 13.531(2) Å, β = 97.006(8)° and *V* = 1245.8(4) Å^3^.

The structure of the previously unknown monoclinic polymorph, B*a*P-II (space group No. 14, *P*2_1_/*c*), was solved and refined at 4.8 GPa [Table S3, Fig. 1 ▸(*b*)] with the unit-cell parameters *a* = 3.59710(10) Å, *b* = 21.658(9) Å, *c* = 12.7908(9) Å, β = 95.339(5)° and *V* = 992.2(4) Å^3^. The arrangement of molecules in B*a*P-II is similar to that in B*a*P-I. In both structures, the molecules display a herringbone pattern projected along the *a* direction [Figs. 1 ▸(*a*) and 1 ▸(*b*), top], but the intermolecular angles in B*a*P-I and B*a*P-II are noticeably different [see the projections along the *b* axis in Figs. 1 ▸(*a*) and 1 ▸(*b*), bottom]. In our previous study of pyrene under pressure (Zhou *et al.*, 2024 ▸), we also noticed that the phase transformation of pyrene-I to pyrene-II is accompanied by a change of the intermolecular angle, whereas the space-group symmetry remains the same.

Further compression of B*a*P-II led to the formation of a new triclinic polymorph, B*a*P-III (space group No. 2, *P*1), which we first observed at 7.1 GPa [Fig. 1 ▸(*c*)]. The unit-cell parameters at 7.1 GPa are as follows: *a* = 3.4912(1) Å, *b* = 12.687(3) Å, *c* = 21.531(6) Å, α = 91.51(2)°, β = 90.434(9)°, γ = 95.820(8)° and *V* = 911.5(2) Å^3^. Table S4 provides detailed crystallographic data for B*a*P-III at six pressure points in the range 7.1–27.9 GPa. As the α and β angles are very close to 90°, the structure of B*a*P-III, if viewed along the *a* and *b* directions, looks very similar to those of B*a*P-I and B*a*P-II (Fig. 1 ▸). However, crystallographically, due to the lower symmetry, the molecular arrangement in B*a*P-III is missing the herringbone pattern. A detailed geometrical analysis of the structures in different polymorphs is given in the *Discussion*[Sec sec4] below.

## 4. Discussion

### 4.1. Compressional behavior of the polymorphs of B*a*P

**Figure 2:**
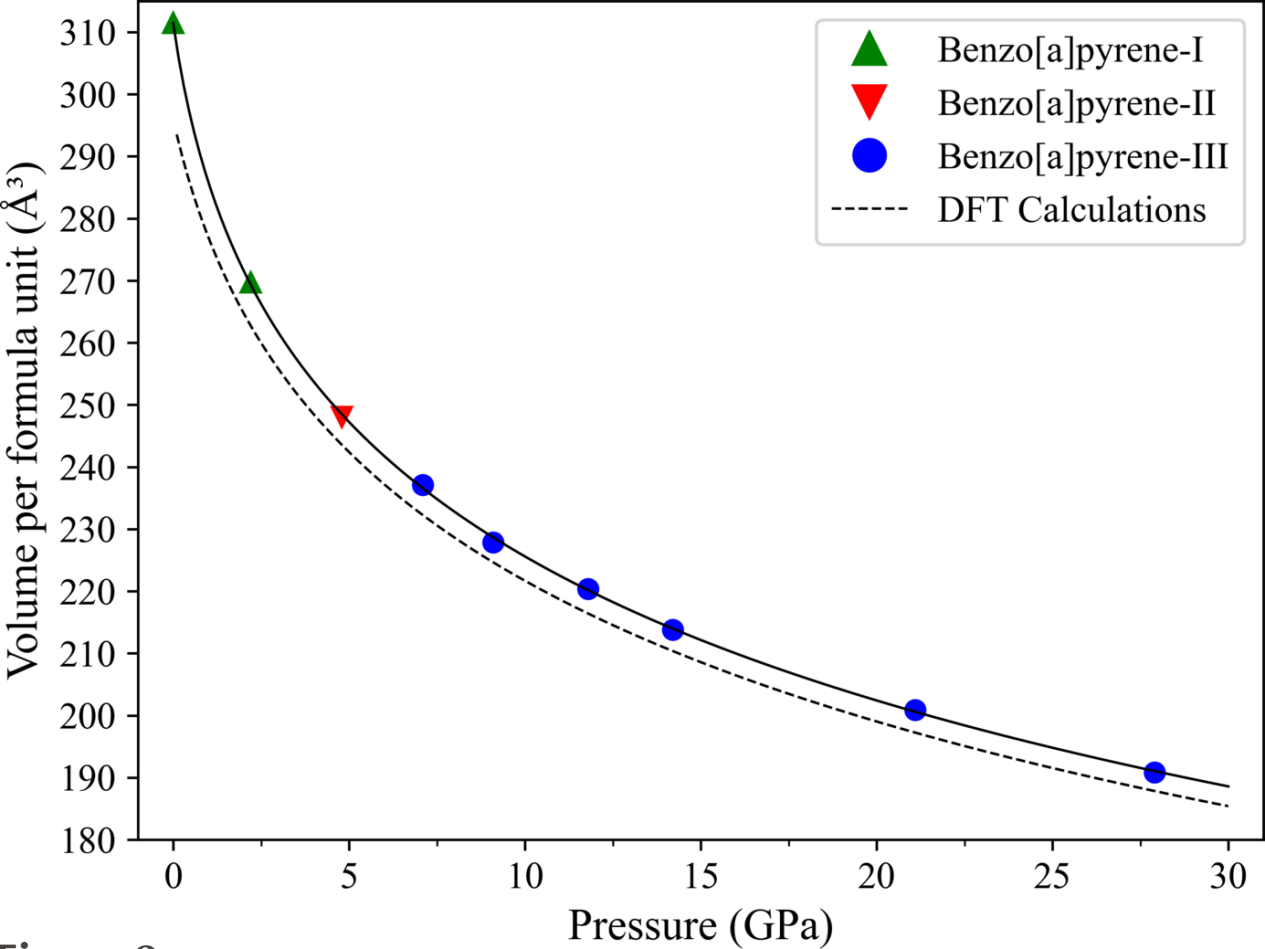
Pressure dependence of the unit-cell volume per formula unit for polymorphs of B*a*P up to 27.9 GPa. The experimental data for B*a*P-I are shown by green solid triangles, for B*a*P-II by red inversed solid triangles and for B*a*P-III by blue solid circles. The solid black line shows the fit of all pressure–volume experimental points using the third-order Birch–Murnaghan EoS with the parameters *V*_0_ = 311.45 Å^3^, *K*_0_ = 7.7(7) GPa and *K*′ = 10.10(10). The dashed black line shows the fit of the calculated pressure–volume data points with the parameters *V*_0_ = 295.92 Å^3^, *K*_0_ = 11.53(14) GPa and *K*′ = 8.28(9).

The compressional behavior of the polymorphs of B*a*P up to 27.9 GPa is presented in Fig. 2 ▸. The values of the unit-cell volume per formula unit for B*a*P-I, B*a*P-II and B*a*P-III as a function of pressure were obtained from our experiments (Table S5). In Fig. 2 ▸ they are shown by solid symbols of different colors. These pressure–volume data were fitted using the third-order Birch–Murnaghan equation of state (EoS) with the volume *V*_0_ = 311.45 Å^3^, which is the volume of B*a*P-I under ambient conditions. The bulk modulus, *K*_0_, and its first derivative, *K*′, were determined to be 7.7(7) GPa and 10.10(10), respectively.

The pressure–volume data points calculated using DFT for each polymorph (Table S6) are consistent with the pressure points at which the phases were observed experimentally. The calculated data have also been fitted using the third-order Birch–Murnaghan EoS. The EoS parameters appeared to be as follows: *V*_0_ = 295.92 Å^3^, *K*_0_ = 11.53(14) GPa and *K*′ = 8.28(9). The volume values from the calculated data fitting are consistently lower than those from the experimental data fitting. This observation can be attributed to the fact that DFT calculations simulate the structures of polymorphs at 0 K, where their volumes are always smaller than those obtained from experiments at room temperature.

**Figure 3:**
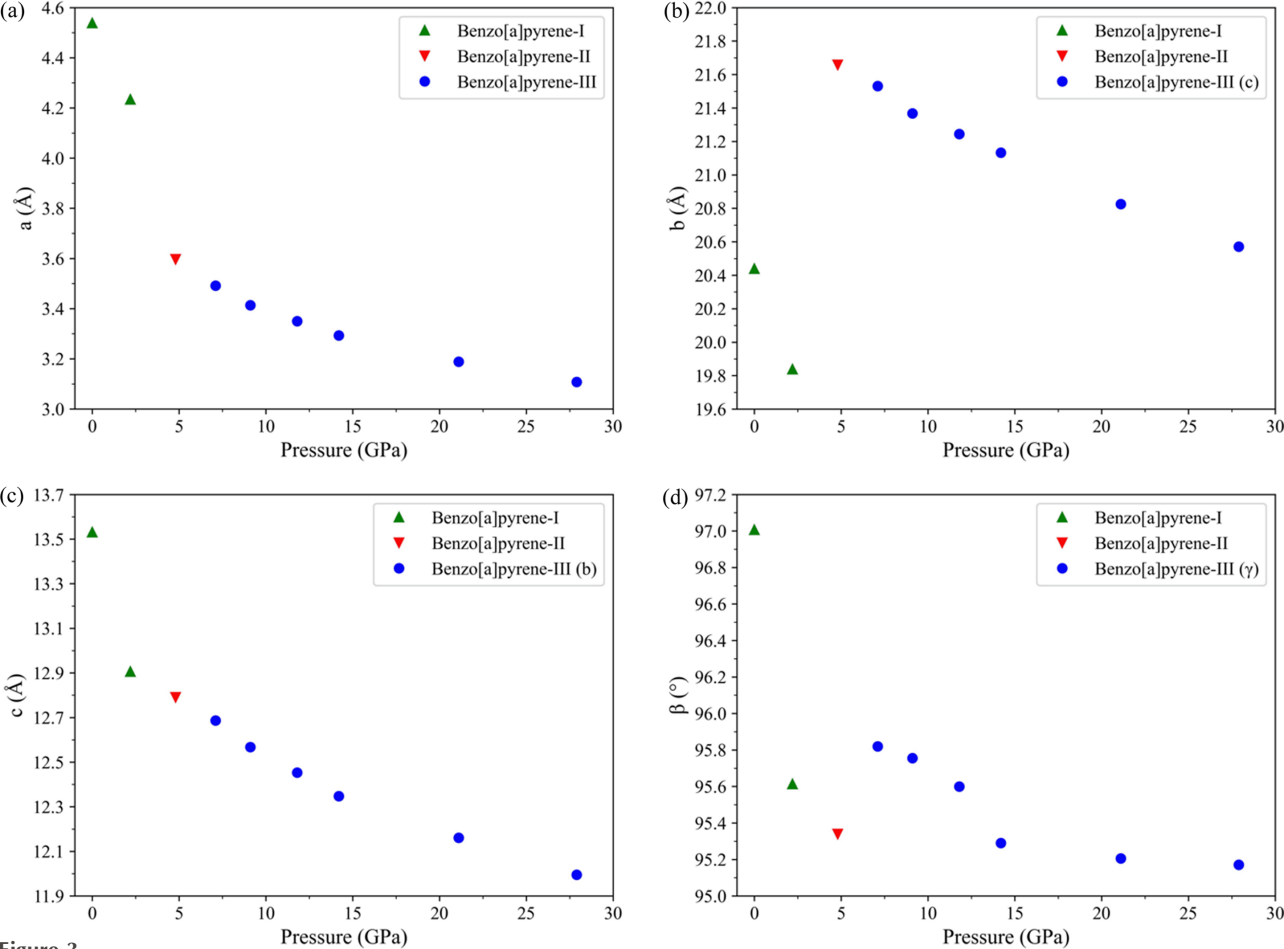
Lattice parameters of B*a*P polymorphs as a function of pressure up to 27.9 GPa. Lattice parameters (*a*) *a*, (*b*) *b*, (*c*) *c* and (*d*) β are designated in the *y* axes for the structures of B*a*P-I and B*a*P-II. For the triclinic structure of B*a*P-III the corresponding lattice parameters are given in brackets in the legend. (As the α and β angles in the triclinic structure of B*a*P-III are very close to 90°, then *b* of B*a*P-I and B*a*P-II corresponds to *c* in B*a*P-III and *vice versa*, while β corresponds to γ in B*a*P-III). Green solid triangles – B*a*P-I, red inversed solid triangles – B*a*P-II and blue solid circles – B*a*P-III.

The dependences of the lattice parameters of B*a*P polymorphs on pressure is shown in Fig. 3 ▸ (see Table S7 for numerical values). On compression of B*a*P-I, the *a* and *b* parameters gradually shorten, but they change substantially during the transition from B*a*P-I to B*a*P-II: the value of the *a* parameter decreases from 4.2338(2) to 3.59710(10) Å, while the *b* parameter increases from 19.838(4) to 21.658(9) Å. The *c* parameter continuously shortens in all phases. The β angle decreases from 97.006(8) to 95.613(5)° on compression of B*a*P-I at pressures still below ∼3 GPa. After a phase transition at 4.8 GPa β slightly rises to 95.820(8)° in B*a*P-III and does not change considerably on compression to the maximum pressure of about 28 GPa.

### 4.2. Theoretical calculations

DFT calculations were conducted at ten pressure points between 1 bar and 40 GPa. Relaxed structural parameters are detailed in Tables S8–S10, which include data for B*a*P-I at ambient pressure, B*a*P-II at 4.8 GPa and B*a*P-III at 7.1 GPa.

**Figure 4:**
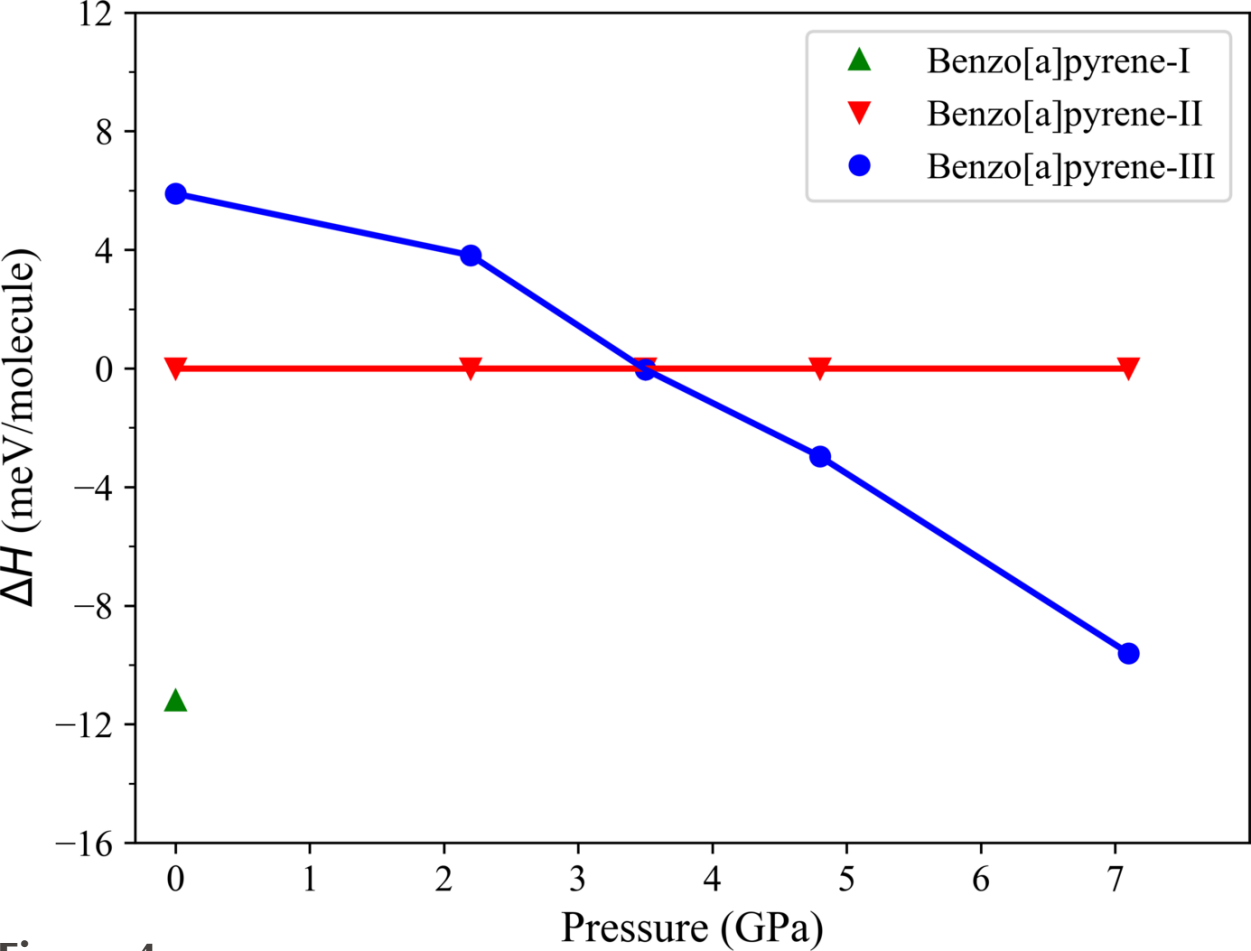
Δ*H* values for the two polymorphs B*a*P-I and B*a*P-III relative to B*a*P-II calculated up to 7.1 GPa. Designations for different polymorphs are as follows: B*a*P-I – green solid triangles, B*a*P-III – blue solid circles and B*a*P-II – red inversed solid triangles.

The enthalpy differences (Δ*H*) for the two polymorphs (B*a*P-I and B*a*P-III) relative to B*a*P-II were calculated as a function of pressure from ambient pressure up to 7.1 GPa at 0 K (Fig. 4 ▸) as described in the *Methods*[Sec sec2]. They revealed that, at ambient pressure, B*a*P-I is relatively more stable than B*a*P-II and B*a*P-III. We found that, at 2.2 GPa, B*a*P-I relaxed into an atomic configuration with an enthalpy much higher than that of B*a*P-II and B*a*P-III. At 3.5 GPa, starting from the atomic configuration of B*a*P-I, atomic positions relaxed to those of B*a*P-II. According to the calculations, B*a*P-III is the most stable phase above 3.5 GPa.

### 4.3. Geometrical analysis of the structures of B*a*P polymorphs

**Figure 5:**
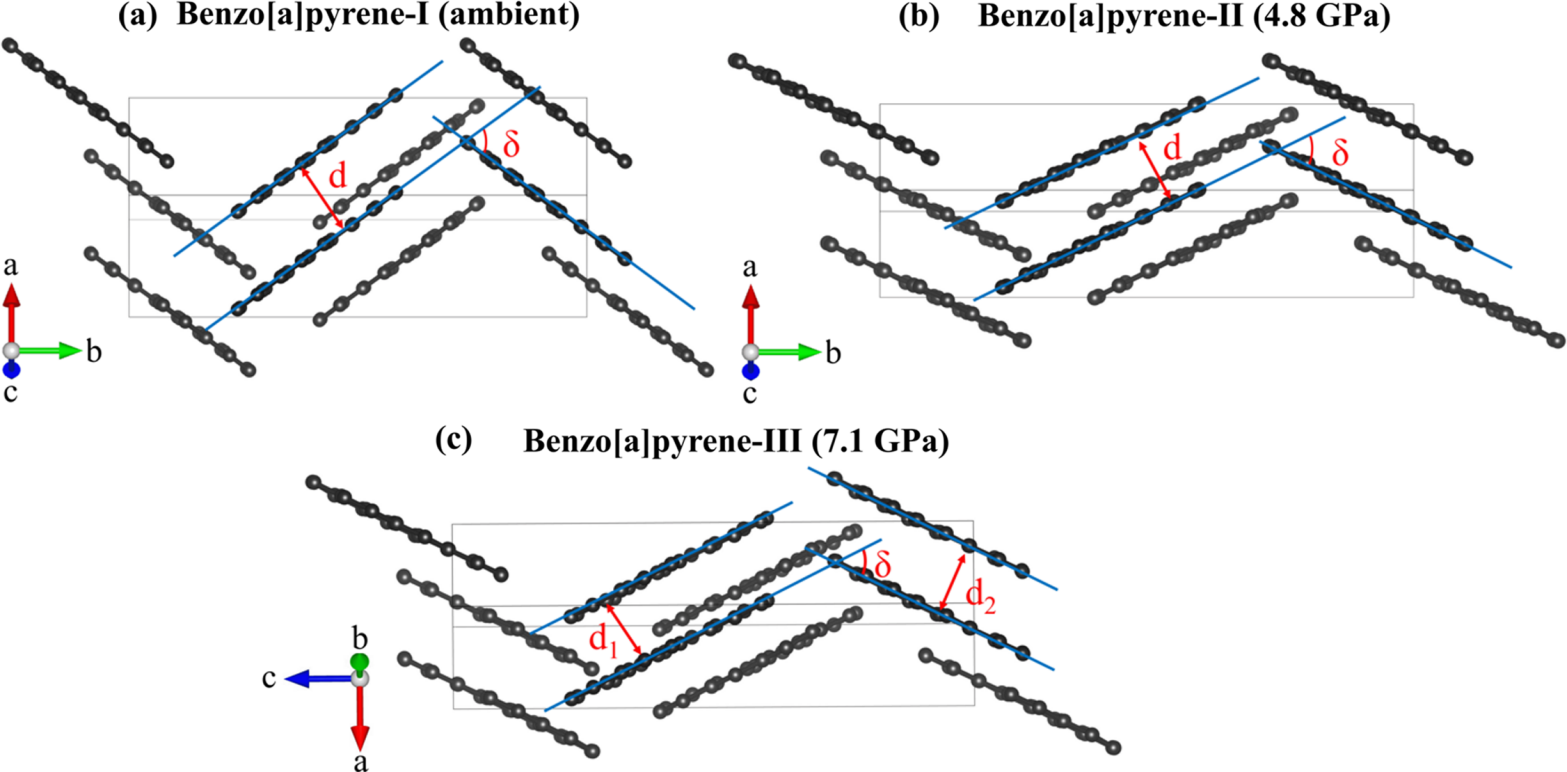
Visualization of intermolecular distances and interplanar angles in the structures of B*a*P polymorphs. (*a*) B*a*P-I and (*b*) B*a*P-II, as viewed along the [504] direction; (*c*) B*a*P-III, as viewed along the [540] direction (hydrogen atoms are not shown, carbon atoms are shown as black dots). The blue lines represent the planes of the flat molecules; in B*a*P-I and B*a*P-II all intermolecular distances are equal (designated *d*); *d*_1_ and *d*_2_ are the interplanar distances in B*a*P-III, which are almost equal; δ is the interplanar angle.

**Figure 6:**
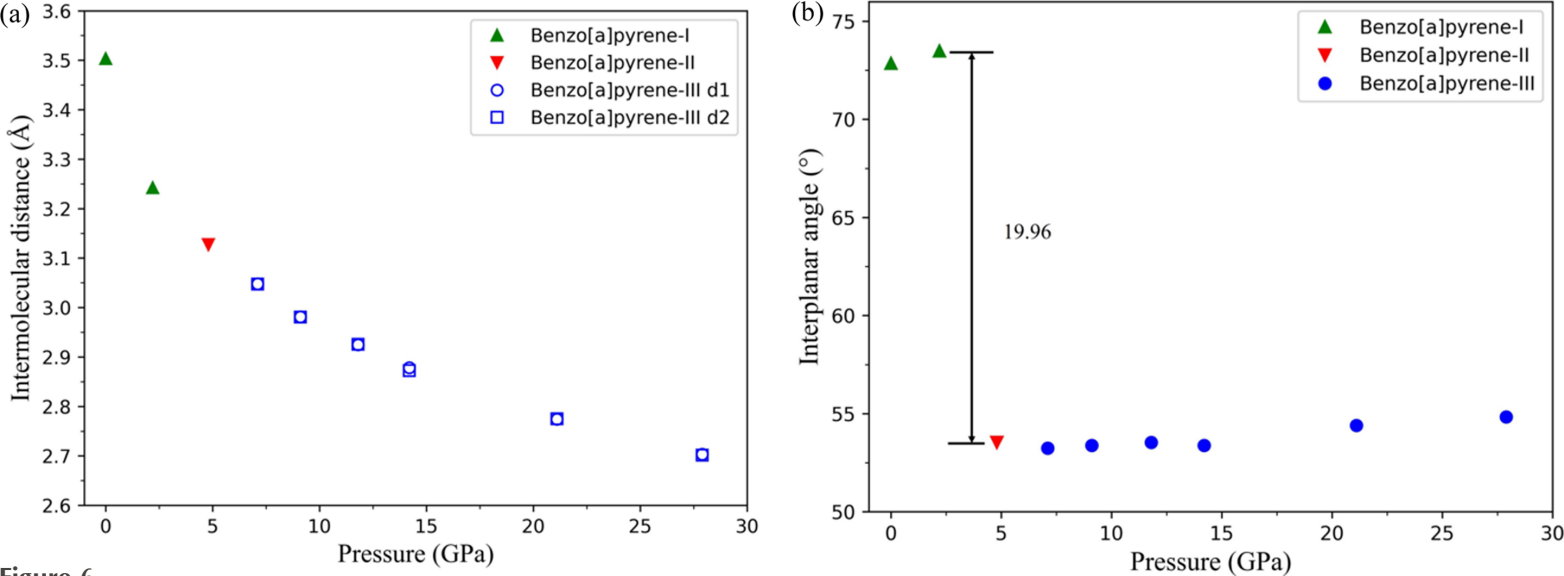
Variation of (*a*) intermolecular distances (see Fig. 5 ▸ for designations) and (*b*) interplanar angles in the B*a*P polymorphs with pressure (see Fig. 5 ▸ for δ).

Fig. 5 ▸ illustrates the structures of B*a*P-I and B*a*P-II viewed along the [540] direction [Figs. 5 ▸(*a*) and 5 ▸(*b*)], and of B*a*P-III viewed along the [504] direction [Fig. 5 ▸(*c*)], chosen to optimally display the topology of the molecular structures of these different polymorphs. To accurately calculate the intermolecular distances in B*a*P polymorphs, the molecules were approximated by mean molecular planes considering 20 carbon atoms in a molecule, using the *NumPy* and *SciPy* libraries in Python (blue lines in Fig. 5 ▸). The intermolecular distances (*d*, *d*_1_ and *d*_2_) and interplanar angles (δ) were calculated using the same software. They are listed in Table S11 and presented graphically in Fig. 6 ▸ as a function of pressure. It is clear that the structure of B*a*P-I undergoes gradual compaction on compression, characterized by a gradual decrease in the intermolecular distances [Fig. 6 ▸(*a*)]. The intermolecular angle in B*a*P-I changes slightly from 72.9° at ambient pressure to 73.5° at 2.2 GPa, whereas in the B*a*P-I to B*a*P-II transformation, the applied stress leads to an abrupt change in the intermolecular angle from 73.5° at 2.2 GPa in B*a*P-I to 53.5° at 4.8 GPa in B*a*P-II. Further compression of B*a*P-II to 7.1 GPa results in a decrease of symmetry to *P*1, indicating the transformation to B*a*P-III. On further compression of B*a*P-III from 4.8 to 27.9 GPa, the angle slowly rises to 54.8°. Interestingly, compared with the behavior of pyrene molecules, which showed curvature with increasing pressure, as found in our previous study of pyrene (Zhou *et al.*, 2024 ▸), in B*a*P polymorphs the molecules remain flat up to about 28 GPa, the highest pressure achieved in this study.

### 4.4. Evolution of intermolecular interactions on compression

**Figure 7:**
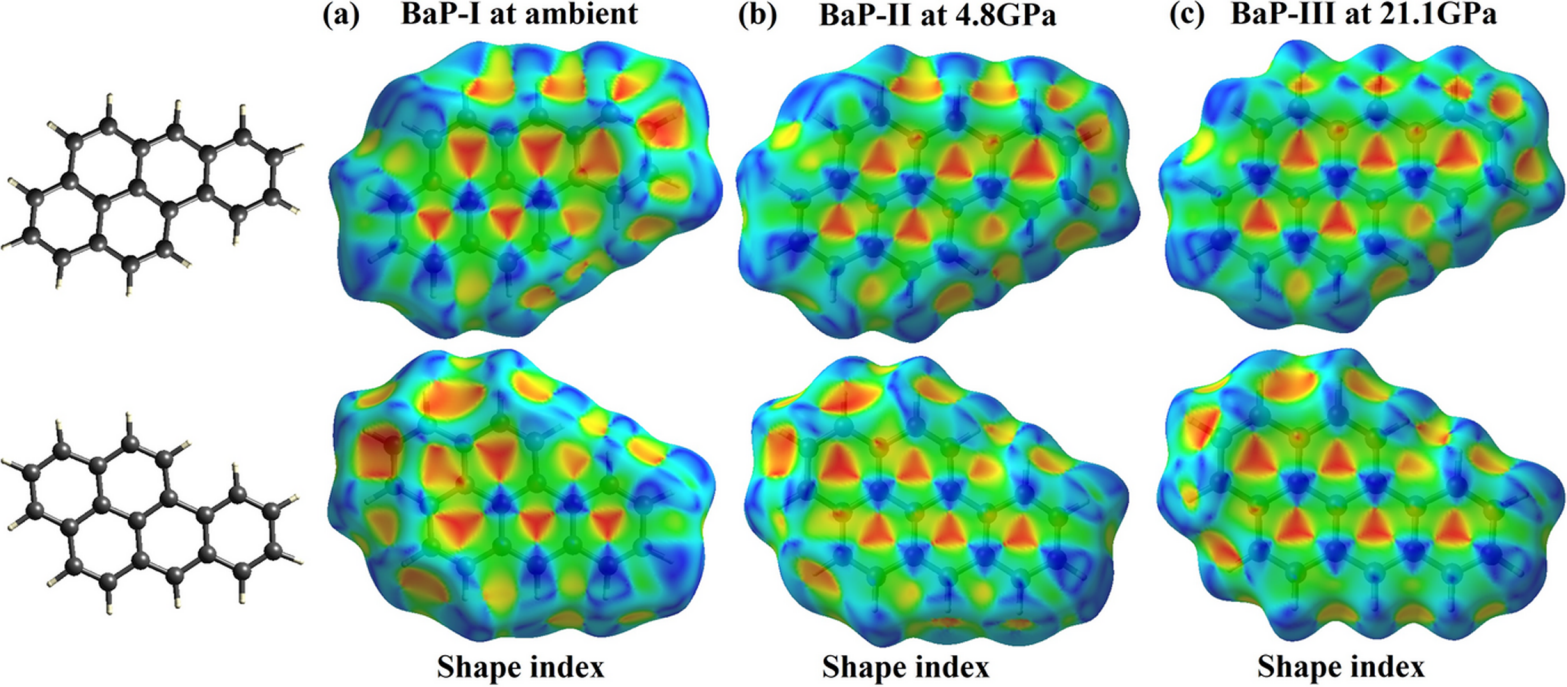
Hirshfeld surfaces of B*a*P polymorphs mapped with the shape index. The front and back views of Hirshfeld surfaces for (*a*) B*a*P-I at ambient pressure, (*b*) B*a*P-II at 4.8 GPa and (*c*) B*a*P-III at 21.1 GPa. Shape index is mapped from −1.0 (red) to 0.0 (green) to 1.0 (blue).

To visualize intermolecular interactions and explore their evolution on compression, we constructed Hirshfeld surfaces for the three B*a*P polymorphs using the *CrystalExplorer* program (Spackman *et al.*, 2021 ▸). Those mapped with the shape index are shown in Fig. 7 ▸. Corresponding fingerprint plots are provided in Fig. S1 of the supporting information. The methodology of the Hirshfeld surface analysis is described in detail in a comprehensive review by McKinnon *et al.* (2004 ▸) and in the paper by Spackman & Jayatilaka (2009 ▸).

The analysis of the Hirshfeld surfaces of B*a*P polymorphs – even based purely on visual inspection – shows that the two sides of a molecule are involved in quite similar contacts with neighboring molecules, participating in a planar stacking arrangement of molecules (π⋯π stacking) showing up as the alternating red and blue triangles in both the front and the back sides of the Hirshfeld surfaces (Fig. 7 ▸) (McKinnon *et al.*, 2004 ▸; Spackman & Jayatilaka, 2009 ▸), as well as a red region near the center of the fingerprint plot (Fig. S1 of the supporting information). These features are quite obvious for all polymorphs (Figs. 7 ▸ and S1). For B*a*P-I the red region in the fingerprint plot is in the vicinity of (*d*_i_, *d*_e_) ≃ 1.8–2.0 Å, a range typical of the interplanar spacing of PAHs (*d*_i_ and *d*_e_ are the distances from the internal or external atoms to the Hirshfeld surface). With increasing pressure this red feature moves to lower (*d*_i_, *d*_e_) values, indicating a decrease in the intermolecular distances [see also Fig. 6 ▸(*a*)]. Blue signifies convex surface curvatures of the Hirshfeld surface corresponding to the direct H⋯H contacts. The red regions of concave curvature reflect the C⋯H interactions between the molecules.

One can break down the Hirshfeld surface into patches associated with specific atom-type/atom-type pairs to highlight just those regions on the surface, and sum the areas of surface patches associated with various contacts (Spackman & Jayatilaka, 2009 ▸). We have made the calculations using the *Crystal­Explorer* program (Spackman *et al.*, 2021 ▸) for all B*a*P polymorphs as a function of pressure (Table S12). As seen, for B*a*P-I the H⋯H interactions are associated with nearly 60% of the surface area, whereas the contribution of the C⋯C interactions is about 20%, and the sum of all C⋯H interactions is also about 20%. Fig. S2 presents percentage contributions to the Hirshfeld surface area for the various close intermolecular contacts (C⋯C, H⋯H, C_e_⋯H_i_ and C_i_⋯H_e_) as a function of pressure for the molecules in the B*a*P polymorphs. The percentage contribution of C⋯C intermolecular contacts increases with pressure, while that of H⋯H contacts decreases, showing an abrupt change as a result of the B*a*P-I to B*a*P-II transition: while the C⋯C contribution still shows an increase (although non-monotonic), the H⋯H contribution also increases, likely as a result of a sharp decrease of the interplanar angle by about 20°. The C⋯H contribution decreases as there is a decrease in the offset of stacked molecules following the B*a*P-I to B*a*P-II transition. However, in the B*a*P-II to B*a*P-III transition, there are no abrupt changes in the percentage contribution of direct H⋯H contacts (which continues to decrease monotonically) while that of the C⋯C contacts increases consistently, and the total C⋯H contribution shows almost no change. The shortest H⋯H contacts considerably decrease in B*a*P-II compared with those in B*a*P-I, but do not noticeably decrease on further compression (see the positions of the sharp features in the left lower corners of the fingerprint plots for B*a*P-I and B*a*P-III in Fig. S1). To summarize, in all three polymorphs, the molecules are involved in similar contacts with neighboring molecules, participating in a planar stacking arrangement. A general trend is observed with an increase in the percentage contribution of C⋯C intermolecular contacts and a decrease in H⋯H contacts on compression.

## 5. Conclusions

We have presented the results of high-pressure studies of B*a*P up to about 28 GPa. These provide insights into the structural transformations in a representative of a broad class of organic materials: PAHs. These studies combined *in situ* synchrotron SC-XRD in a diamond anvil cell and *ab initio* calculations. At 4.8 GPa, B*a*P-I was found to transform to a previously unknown B*a*P-II phase with the same space group (*P*2_1_/*c*). The transformation manifests as an abrupt change in the intermolecular angle and unit-cell parameters. The second previously unknown polymorph, B*a*P-III, was detected at 7.1 GPa and preserved under an He pressure medium up to 27.9 GPa. According to the DFT calculations, B*a*P-III is the most stable phase above 3.5 GPa.

## 6. Related literature

The following references are cited in the supporting information: Carrell *et al.* (1997 ▸).

## Supplementary Material

Crystal structure: contains datablock(s) benzoapyrene_0GPa, benzoapyrene_2.2GPa, benzoapyrene_4.8GPa, benzoapyrene_7.1GPa, benzoapyrene_9.1GPa, benzoapyrene_11.8GPa, benzoapyrene_14.2GPa, benzoapyrene_21.1GPa, benzoapyrene_27.9GPa. DOI: 10.1107/S2052252524010455/lq5058sup1.cif

Structure factors: contains datablock(s) benzoapyrene_0GPa. DOI: 10.1107/S2052252524010455/lq5058benzoapyrene_0GPasup2.hkl

Structure factors: contains datablock(s) benzoapyrene_2.2GPa. DOI: 10.1107/S2052252524010455/lq5058benzoapyrene_2.2GPasup3.hkl

Structure factors: contains datablock(s) benzoapyrene_4.8GPa. DOI: 10.1107/S2052252524010455/lq5058benzoapyrene_4.8GPasup4.hkl

Structure factors: contains datablock(s) benzoapyrene_7.1GPa. DOI: 10.1107/S2052252524010455/lq5058benzoapyrene_7.1GPasup5.hkl

Structure factors: contains datablock(s) benzoapyrene_9.1GPa. DOI: 10.1107/S2052252524010455/lq5058benzoapyrene_9.1GPasup6.hkl

Structure factors: contains datablock(s) benzoapyrene_11.8GPa. DOI: 10.1107/S2052252524010455/lq5058benzoapyrene_11.8GPasup7.hkl

Structure factors: contains datablock(s) benzoapyrene_14.2GPa. DOI: 10.1107/S2052252524010455/lq5058benzoapyrene_14.2GPasup8.hkl

Structure factors: contains datablock(s) benzoapyrene_21.1GPa. DOI: 10.1107/S2052252524010455/lq5058benzoapyrene_21.1GPasup9.hkl

Structure factors: contains datablock(s) benzoapyrene_27.9GPa. DOI: 10.1107/S2052252524010455/lq5058benzoapyrene_27.9GPasup10.hkl

Supporting figures and tables. DOI: 10.1107/S2052252524010455/lq5058sup11.pdf

CCDC references: 2360829, 2360830, 2360831, 2360832, 2360833, 2360834, 2360835, 2360836, 2360837
