# Supplementary material for: Structural transformations and stability of benzo[a]pyrene under high pressure
Source: IUCrJ. 2025 Jan 1;12(Pt 1):16–22. doi: 10.1107/S2052252524010455 (PMC11707690; doi:10.1107/S2052252524010455)
Supplement: Supplementary file 11 [file m-12-00016-sup11.pdf]

IUCrJ

Volume 12 (2025)

Supporting information for article:

Structural transformations and stability of benzo[*a*]pyrene under high pressure

Wenju Zhou, Andrey Aslandukov, Anastasiia Minchenkova, Michael Hanfland, Leonid Dubrovinsky and Natalia Dubrovinskaia

Table S1 Summary of the experiments conducted in this work.

DAC number	DAC type	Anvils type/ culet size, μm	Starting material/ pressure transmitting medium	Beamline/XRD wavelength, Å	Result	Pressure, GPa
1	Membrane-type	Boehler-Almax 250	B*a*P-I/ No medium	ID15B ESRF, 0.4100	B*a*P-I	ambient
2	Membrane-type	Boehler-Almax 250	B*a*P-I/ Helium	ID15B ESRF, 0.4100	B*a*P-I<br>B*a*P-II<br>B*a*P-III	2.2(3)<br>4.8(3)<br>7.1(3)<br>9.1(3)<br>11.8(3)<br>14.2(4)<br>21.1(3)<br>27.9(4)

Table S2 Experimental crystallographic data for B*a*P-I obtained by single-crystal X-ray diffraction at room temperature, at ambient pressure and at 2.2 GPa in this work and at low temperature at ambient pressure in ref. (Carrell et al., 1997)

	B*a*P-I at ambient	B*a*P-I at 2.2 GPa	B*a*P-I at 120 K
CCDC deposition number	2360829	2360830	1830498
Crystal data			
Chemical formula	$C_{20}H_{12}$	$C_{20}H_{12}$	$C_{20}H_{12}$
M_r	252.30	252.30	252.30
Crystal system, space group	Monoclinic, $P2_1/c$	Monoclinic, $P2_1/c$	Monoclinic, $P2_1/c$
a, b, c (Å)	4.5384(3), 20.439(5), 13.531(2)	4.2338(2), 19.838(4), 12.9059(10)	4.489(1), 20.309(6), 13.372(5)
α, β, γ (°)	90, 97.006(8), 90	90, 95.613(5), 90	90, 96.59(2), 90
V (Å^3)	1245.8(4)	1078.8(2)	1211.0(6)
Z	4	4	4
Density (Mg/m^3)	1.345	1.553	1.384
Wavelength (Å)	0.4100	0.4100	0.7107
μ (mm^{-1})	0.035	0.040	0.08
Data collection			
Absorption correction	Multi-scan	Multi-scan	
T_{min}, T_{max}	0.30, 1.00	0.08, 1.00	
No. of measured, independent and observed reflections	3094, 1877, 661	2644, 1646, 831	
R_{int}	0.035	0.013	
θ_{max} (°)	21.63	21.35	
Refinement			
Refinement on	F^2	F^2	
$R[F^2>2\sigma(F^2)]$, $wR(F^2)$, S	0.060, 0.145, 0.901	0.069, 0.218, 0.978	

Data / restraints / parameters	1877/ 0/ 181	1646/ 0/ 181	
H-atom treatment	Refined by ride model	Refined by ride model	
Weighting scheme	$w = 1/[\sigma^2 (F_o^2) + (0.0949P)^2]$, where $P= (F_o^2+2F_c^2)/3$	$w = 1/[\sigma^2 (F_o^2) + (0.1566P)^2]$, where $P= (F_o^2+2F_c^2)/3$	$w = 1/[\sigma^2 (F_o^2)]$
$\Delta\rho_{max}$, $\Delta\rho_{min}$ (e Å^{-3})	0.08, -0.10	0.25, -0.20	

Table S3 Experimental crystallographic data for B*a*P-II obtained by single-crystal X-ray diffraction at room temperature at 4.8 GPa in this work.

	B*a*P-II at 4.8 GPa
CCDC deposition number	2360831
Crystal data	
Chemical formula	$C_{20}H_{12}$
M_r	252.30
Crystal system, space group	Monoclinic, $P2_1/c$
a, b, c (Å)	3.59710(10), 21.658(9), 12.7908(9)
α, β, γ (°)	90, 95.339(5), 90
V (Å^3)	992.2(4)
Z	4
Density (Mg/m^3)	1.689
Wavelength (Å)	0.4100
μ (mm^{-1})	0.044
Data collection	
Absorption correction	Multi-scan
T_{min}, T_{max}	0.19, 1.00
No. of measured, independent and observed reflections	2486, 1313, 796
R_{int}	0.020
θ_{max} (°)	21.49
Refinement	
Refinement on	F^2
R[F^2>2σ(F^2)], wR(F^2), S	0.052, 0.141, 0.959
Data / restraints / parameters	1313/ 0/ 181
H-atom treatment	Refined by ride model

Weighting scheme	$w = 1/[\sigma^2 (F_o^2) + (0.1059P)^2]$, where $P = (F_o^2 + 2F_c^2)/3$
$\Delta\rho_{max}$, $\Delta\rho_{min}$ (e Å^{-3})	0.20, -0.16

Table S4 Experimental crystallographic data for BaP-III obtained by single-crystal X-ray diffraction at room temperature and a series of pressures in this work.

	B*a*P-III at 7.1 GPa	B*a*P-III at 9.1 GPa
CCDC deposition number	2360832	2360833
Crystal data		
Chemical formula	$C_{20}H_{12}$	$C_{20}H_{12}$
M_r	252.30	252.30
Crystal system, space group	Triclinic, *P*-1	Triclinic, *P*-1
a, b, c (Å)	3.49120(10), 12.687(3), 21.531(6)	3.41340(10), 12.5674(19), 21.367(5)
α, β, γ (°)	91.51(2), 90.434(9), 95.820(8)	91.675(15), 90.433(7), 95.755(6)
V ($Å^3$)	948.4(3)	911.5(2)
Z	4	4
Density (Mg/m^3)	1.767	1.838
Wavelength (Å)	0.4100	0.4100
μ (mm^{-1})	0.046	0.048
Data collection		
Absorption correction	Multi-scan	Multi-scan
T_{min}, T_{max}	0.16, 1.00	0.36, 1.00
No. of measured, independent and observed reflections	2260, 1531, 867	2374, 1617, 1036
R_{int}	0.031	0.017
θ_{max} (°)	20.99	21.30
Refinement		
Refinement on	F^2	F^2
$R[F^2>2\sigma(F^2)]$,$wR(F^2)$, S	0.063, 0.142, 0.957	0.051, 0.129, 0.949
Data / restraints / parameters	1531/ 66/ 361	1617/ 24/ 361

H-atom treatment	Refined by ride model	Refined by ride model
Weighting scheme	$w = 1/[\sigma^2(F_o^2) + (0.0973P)^2]$,	$w = 1/[\sigma^2 (F_o^2) + (0.1029P)^2]$,
	where $P = (F_o^2 + 2F_c^2)/3$	where $P= (F_o^2+2F_c^2)/3$
$\Delta\rho_{max}$, $\Delta\rho_{min}$ (e Å^{-3})	0.20, -0.15	0.18, -0.19

Table S4 (continuation)

B*a*P-III at 11.8 GPa	B*a*P-III at 14.2GPa	B*a*P-III at 21.1 GPa	B*a*P-III at 27.9 GPa
2360834	2360835	2360836	2360837
$C_{20}H_{12}$	$C_{20}H_{12}$	$C_{20}H_{12}$	$C_{20}H_{12}$
252.30	252.30	252.30	252.30
Triclinic, *P*-1	Triclinic, *P*-1	Triclinic, P-1	Triclinic, P-1
3.35010(10), 12.453(2), 21.244(5)	3.2931(2), 12.347(4), 21.133(7)	3.1883(4), 12.1605(18), 20.825(6)	3.1077(4), 11.995(2), 20.571(8)
91.709(18), 90.417(8), 95.599(7)	91.42(3), 90.439(11), 95.290(11)	91.99(2), 90.297(18), 95.206(11)	91.56(3), 89.92(3), 95.171(13)
881.6(3)	855.3(4)	803.6(3)	763.4(3)
4	4	4	4
1.901	1.959	2.085	2.195
0.4100	0.4100	0.4100	0.4100
0.049	0.051	0.054	0.057
Multi-scan	Multi-scan	Multi-scan	Multi-scan
0.04, 1.00	0.02, 1.00	0.06, 1.00	0.04, 1.00
2203, 1482, 979	2119, 1448, 790	1711, 1242, 801	988, 764, 431
0.023	0.022	0.023	0.059
21.32	21.30	21.17	15.86
F^2	F^2	F^2	F^2
0.057, 0.152, 0.972	0.076, 0.199, 1.005	0.063, 0.184, 0.986	0.143, 0.324, 1.677
1482/ 78/ 361	1448/ 90/ 361	1242/ 72/ 361	764/ 186/ 361
Refined by ride model	Refined by ride model	Refined by ride model	Refined by ride model

$w = 1/[\sigma^2(F_o^2) + (0.1282P)^2]$, where $P = (F_o^2 + 2F_c^2)/3$	$w = 1/[\sigma^2 (F_o^2) + (0.1529P)^2]$, where $P = (F_o^2 + 2F_c^2)/3$	$w = 1/[\sigma^2(F_o^2) + (0.1784P)^2]$, where $P = (F_o^2 + 2F_c^2)/3$	$w = 1/[\sigma^2(F_o^2) + (0.2P)^2]$, where $P = (F_o^2 + 2F_c^2)/3$
0.21, -0.18	0.23, -0.20	0.18, -0.16	0.31, -0.34

Table S5 Unit cell volume per formula unit for B*a*P polymorphs up to 27.9 GPa in this work.

Polymorph/ pressure transmitting medium	Pressure, GPa	Volume per formula unit, Å^3
B*a*P-I/ No medium	0	311.45(10)
B*a*P-I/ He	2.2(3)	269.70(5)
B*a*P-II/ He	4.8(3)	248.05(10)
B*a*P-III/ He	7.1(3)	237.10(8)
B*a*P-III/ He	9.1(3)	227.88(5)
B*a*P-III/ He	11.8(3)	220.40(8)
B*a*P-III/ He	14.2(4)	213.83(10)
B*a*P-III/ He	21.1(3)	200.90(8)
B*a*P-III/ He	27.9(4)	190.85(8)

Table S6 DFT-calculated unit cell volume per formula unit of B*a*P polymorphs up to 40 GPa.

Polymorph	Pressure (GPa)	Volume per formula unit (Å^3)
B*a*P-I	0	295.92
B*a*P-I	2.2	263.06
B*a*P-II	4.8	243.28
B*a*P-III	7.1	231.74
B*a*P-III	11.8	216.64
B*a*P-III	14.2	210.16
B*a*P-III	21.1	197.29
B*a*P-III	27.9	187.86
B*a*P-III	40	175.72

Table S7 Lattice parameters of B*a*P polymorphs up to 35.5 GPa determined in this work.

Polymorph/ pressure transmitting medium	Pressure, GPa	a, Å	b, Å	c, Å	α, °	β, °	γ, °
B*a*P-I/No medium	0	4.5384(3)	20.439(5)	13.531(2)	90	97.006(8)	90
B*a*P-I/ He	2.2(3)	4.2338(2)	19.838(4)	12.9059(1)	90	95.613(5)	90
B*a*P-II/ He	4.8(3)	3.5971(1)	21.658(9)	12.7908(9)	90	95.339(5)	90
B*a*P-III/ He	7.1(3)	3.4912(1)	12.687(3)	21.531(6)	91.51(2)	90.434(9)	95.820(8)
B*a*P-III/ He	9.1(3)	3.4134(1)	12.5674(19)	21.367(5)	91.675(15)	90.433(7)	95.755(6)
B*a*P-III/ He	11.8(3)	3.3501(1)	12.453(2)	21.244(5)	91.709(18)	90.417(8)	95.599(7)
B*a*P-III/ He	14.2(4)	3.2931(2)	12.347(4)	21.133(7)	91.42(3)	90.439(11)	95.290(11)
B*a*P-III/ He	21.1(3)	3.1883(4)	12.1605(18)	20.825(6)	91.99(2)	90.297(18)	95.206(11)
B*a*P-III/ He	27.9(4)	3.1077(4)	11.995(2)	20.571(8)	91.56(3)	89.92(3)	95.171(13)

Table S8a DFT-calculated crystallographic data for B*a*P-I at ambient pressure.

B*a*P-I	
Chemical formula	$C_{20}H_{12}$
M_r	252.30
Crystal system, space group	Monoclinic, $P2_1/c$
a, b, c (Å)	4.444, 20.315, 13.195
α, β, γ (°)	90, 96.396,90
V (Å^3)	1183.690
Z	4

Table S8b Theoretical atomic coordinates for B*a*P-I at ambient pressure.

Label	x	y	z
C1	0.6790	0.4583	0.6470
C2	0.2430	0.6537	0.5536
C3	0.3121	0.6115	0.7308
C4	0.5244	0.6632	0.7563
C5	0.2424	0.5656	0.8060
C6	0.5900	0.6233	0.9325
C7	0.5948	0.7092	0.6804
C8	0.8901	0.5094	0.6748
C9	0.1693	0.6064	0.6277
C10	0.0304	0.5134	0.7781
C11	0.3868	0.5737	0.9074
C12	0.9631	0.5562	0.6023
C13	0.6678	0.6693	0.8581
C14	0.9531	0.4648	0.8478
C15	0.8799	0.7201	0.8819
C16	0.4469	0.7027	0.5783
C17	0.7480	0.4154	0.8185
C18	0.8063	0.7591	0.7076
C19	0.9482	0.7642	0.8073

C20	0.6076	0.4122	0.7170
H1	0.1299	0.6491	0.4763
H2	0.5027	0.7379	0.5207
H3	0.0582	0.4660	0.9264
H4	0.6927	0.3788	0.8739
H5	0.8628	0.7940	0.6498
H6	0.6977	0.6281	0.0107
H7	0.3346	0.5398	0.9669
H8	0.5747	0.4562	0.5683
H9	0.8549	0.5526	0.5241
H10	0.9920	0.7240	0.9595
H11	0.4427	0.3736	0.6951
H12	0.1155	0.8028	0.8256

Table S9a Theoretical crystallographic data for B*a*P-II at 4.8 GPa.

B*a*P-II	
Chemical formula	$C_{20}H_{12}$
M_r	252.30
Crystal system, space group	Monoclinic, $P2_1/c$
a, b, c (Å)	3.537, 21.750, 12.695
α, β, γ (°)	90, 94.906, 90
V (Å^3)	973.124
Z	4

Table S9b Theoretical atomic coordinates for B*a*P-II at 4.8 GPa.

Label	x	y	z
C1	0.9537	0.4918	0.2184
C2	0.4973	0.3363	0.2351
C3	0.6992	0.3909	0.2639
C4	0.0468	0.4521	0.3997
C5	0.7517	0.4368	0.1873
C6	0.7902	0.3520	0.4469
C7	0.4095	0.3734	0.0532
C8	0.3502	0.3272	0.1287
C9	0.1017	0.4987	0.3260
C10	0.3051	0.5526	0.3568
C11	0.2096	0.5923	0.1801
C12	0.2403	0.2371	0.2816
C13	0.8489	0.3991	0.3715
C14	0.3600	0.5985	0.2858
C15	0.1463	0.2734	0.1017
C16	0.0955	0.2288	0.1770
C17	0.5881	0.3007	0.4191
C18	0.6034	0.4260	0.0814

C19	0.4397	0.2905	0.3124
C20	0.0121	0.5401	0.1475
H1	0.1593	0.4584	0.4813
H2	0.9128	0.3590	0.5274
H3	0.2942	0.3663	0.9719
H4	0.4168	0.5568	0.4387
H5	0.2528	0.6288	0.1237
H6	0.1950	0.2020	0.3403
H7	0.5264	0.6388	0.3101
H8	0.0264	0.2675	0.0207
H9	0.9324	0.1878	0.1559
H10	0.5351	0.2662	0.4778
H11	0.6449	0.4602	0.0214
H12	0.8967	0.5365	0.0658

Table S10a Theoretical crystallographic data for B*a*P-III at 7.1 GPa.

B*a*P-III	
Chemical formula	$C_{20}H_{12}$
M_r	252.30
Crystal system, space group	Triclinic, *P*-1
a, b, c (Å)	3.434, 12.608, 21.532
α, β, γ (°)	91.558, 90.403, 95.870
V (Å^3)	926.940
Z	4

Table S10b Theoretical atomic coordinates for B*a*P-III at 7.1 GPa.

Label	x	y	z
C1	0.9737	0.2128	0.4938
C2	0.0462	0.2785	0.9915
C3	0.5217	0.2449	0.3370
C4	0.5244	0.2695	0.8356
C5	0.7295	0.2685	0.3936
C6	0.3165	0.2371	0.8895
C7	0.1062	0.3977	0.4615
C8	0.9709	0.0960	0.9478
C9	0.7678	0.1874	0.4366
C10	0.2520	0.3128	0.9370
C11	0.8464	0.4557	0.3630
C12	0.2402	0.0531	0.8460
C13	0.4083	0.0586	0.3660
C14	0.5889	0.4527	0.8764
C15	0.3607	0.1389	0.3227
C16	0.6626	0.3782	0.8288
C17	0.1448	0.3194	0.5054
C18	0.9053	0.1688	0.9961
C19	0.3546	0.3447	0.5613

C20	0.6975	0.1351	0.0495
C21	0.2144	0.1651	0.5944
C22	0.7814	0.3129	0.0935
C23	0.2689	0.3011	0.2389
C24	0.8076	0.2277	0.7361
C25	0.8992	0.3753	0.4067
C26	0.1743	0.1272	0.8952
C27	0.3864	0.2697	0.6052
C28	0.6326	0.2051	0.0971
C29	0.1522	0.1171	0.2670
C30	0.8722	0.4091	0.7758
C31	0.1126	0.1968	0.2254
C32	0.9430	0.3349	0.7300
C33	0.6248	0.4336	0.3109
C34	0.4447	0.0850	0.7949
C35	0.6033	0.0824	0.4209
C36	0.3908	0.4211	0.9285
C37	0.4695	0.3271	0.2948
C38	0.5943	0.1937	0.7880
C39	0.0145	0.1373	0.5398
C40	0.9820	0.3482	0.0416
H1	0.2360	0.4782	0.4721
H2	0.8647	0.0128	0.9524
H3	0.9817	0.5359	0.3734
H4	0.1201	0.9706	0.8507
H5	0.2821	0.9776	0.3550
H6	0.6952	0.5359	0.8706
H7	0.4892	0.4253	0.5694
H8	0.5919	0.0515	0.0523
H9	0.2455	0.1054	0.6291
H10	0.7380	0.3680	0.1320
H11	0.2308	0.3634	0.2061

H12	0.8671	0.1697	0.6999
H13	0.5524	0.2902	0.6475
H14	0.4605	0.1780	0.1367
H15	0.0188	0.0367	0.2573
H16	0.9822	0.4924	0.7718
H17	0.9462	0.1793	0.1828
H18	0.1113	0.3588	0.6895
H19	0.5623	0.4965	0.2801
H20	0.4970	0.0284	0.7579
H21	0.6350	0.0194	0.4532
H22	0.3370	0.4804	0.9639
H23	0.8827	0.0560	0.5326
H24	0.0964	0.4312	0.0396

Table S11 Intermolecular distances and interplanar angles of B*a*P polymorphs up to 27.9 GPa from experiments in helium pressure medium.

Polymorph	Pressure, GPa	Intermolecular distance d_1, Å	Intermolecular distance d_2, Å	Interplanar angle, °
B*a*P-I	0	3.503	3.503	72.85
B*a*P-I	2.2	3.242	3.242	73.49
B*a*P-II	4.8	3.127	3.127	53.53
B*a*P-III	7.1	3.048	3.047	53.24
B*a*P-III	9.1	2.981	2.981	53.38
B*a*P-III	11.8	2.925	2.926	53.53
B*a*P-III	14.2	2.878	2.873	53.38
B*a*P-III	21.1	2.774	2.775	54.40
B*a*P-III	27.9	2.703	2.701	54.83

Polymorph	Pressure, GPa	C…C, %	H…H, %	C_e…H_i, %	C_i…H_e, %
B*a*P-I	0	20.9	57.9	7.6	13.6
B*a*P-I	2.2	22.4	54.2	8.4	15.1
B*a*P-II	4.8	27.3	55.7	5.4	11.6
B*a*P-III	7.1	27.6	54.6	5.7	12.1
B*a*P-III	9.1	28.5	53.9	5.6	12.0
B*a*P-III	11.8	29.0	53.3	5.5	12.1
B*a*P-III	14.2	29.8	52.8	5.5	11.9
B*a*P-III	21.1	30.5	51.5	5.6	12.5

Table S12 Percentage contributions to the Hirschfeld surface area for the various close intermolecular contacts (C···C, H···H, C_e···H_i, C_i···H_e) as a function of pressure for molecules in B*a*P polymorphs.

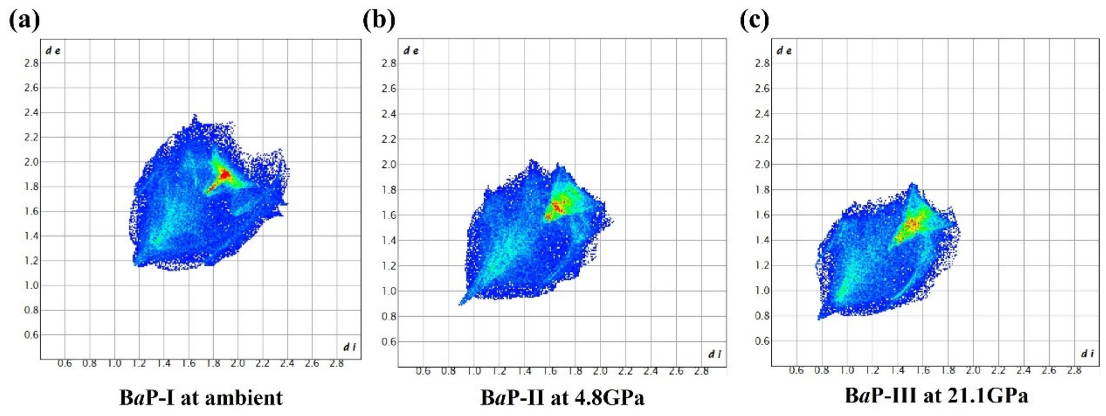

Figure S1 Fingerprint plots for B*a*P polymorphs. (a) B*a*P-I molecule at ambient, (b) B*a*P-II molecule at 4.8 GPa, and (c) B*a*P-III molecule at 21.1GPa.

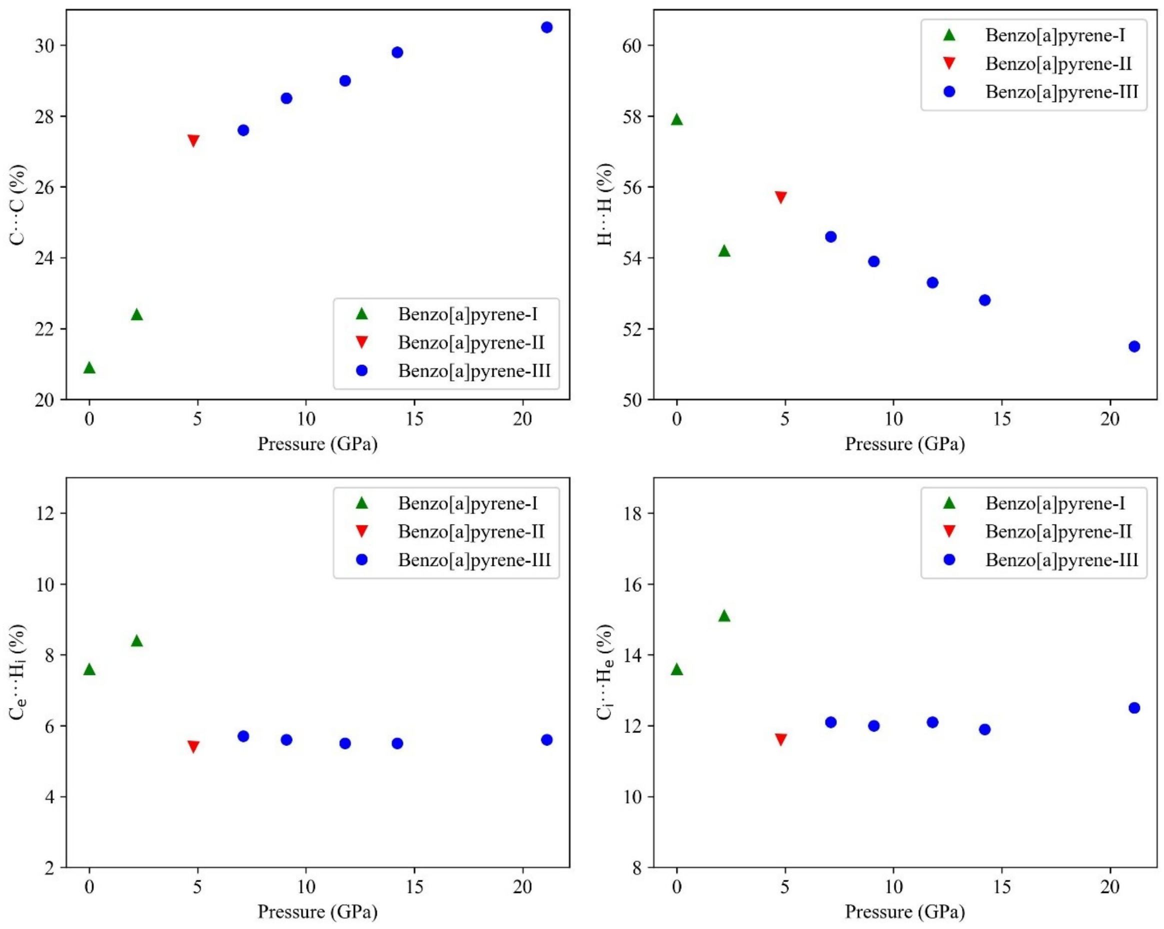

Figure S2 Percentage contribution to the Hirschfeld surface area for the various close intermolecular contacts (C···C, H···H, $C_e \cdots H_i$, $C_i \cdots H_e$) as a function of pressure for molecules in B*a*P polymorphs.
